# Supplementary material for: Cryo-EM structure of the yeast TREX complex and coordination with the SR-like protein Gbp2
Source: eLife. 2021 Mar 31;10:e65699. doi: 10.7554/eLife.65699 (PMC8043747; doi:10.7554/eLife.65699)
Supplement: Supplementary file 1. [file elife-65699-supp1.docx]

**Table 1. Cryo-EM data collection, refinement and validation statistics**

|  | THO-Sub2  protomer  (EMD-23527)  (PDB 7LUV) | THO-Sub2  tetramer  (EMD-23527) |
| --- | --- | --- |
| **Data collection and processing** |  |  |
| Microscope/Camera | Titan Krios/Falcon 3EC | |
| Voltage (kV) | 300 | |
| Electron exposure (e–/Å^2^) | 50 | |
| Defocus range (μm) | 0.8 to 2.0 | |
| Pixel size (Å) | 0.681 | |
| Symmetry imposed | C1 | C2 |
| Initial particle images (no.) | 396 K | |
| Final particle images (no.) | 30 K | 15 K |
| Resolution at 0.143 FSC (masked, Å) | 3.70 | 4.80 |
| Map sharpening *B* factor (Å^2^) | 86 | 145 |
|  |  |  |
| **Refinement** |  |  |
| Model resolution at 0.5 FSC (Å) | 3.86 |  |
| Model composition  Protein residues | 2378 |  |
| *B* factors (Å^2^)  Protein | 114.5 |  |
| R.m.s. deviations  Bond lengths (Å)  Bond angles (°) | 0.008  1.08 |  |
| Validation  MolProbity score  Clashscore  Poor rotamers (%) | 2.08  10.69  0.05 |  |
| Ramachandran plot  Favored (%)  Allowed (%)  Disallowed (%) | 90.4  9.3  0.3 |  |
